# Hyperemesis gravidarum and pregnancy outcomes in the Norwegian mother and child cohort – a cohort study

**DOI:** 10.1186/1471-2393-13-169

**Published:** 2013-09-03

**Authors:** Åse V Vikanes, Nathalie C Støer, Per Magnus, Andrej M Grjibovski

**Affiliations:** 1 Department for Genes and Environment, Division for Epidemiology, Norwegian Institute of Public Health, Nydalen, P.O. Box 4404, Oslo 0403, Norway; 2 Department of Mathematics, University of Oslo, Oslo, Norway; 3 Department for International Public Health, Norwegian Institute of Public Health, Oslo, Norway; 4 International School of Public Health, Northern State Medical University, Arkhangelsk, Russia

**Keywords:** Hyperemesis gravidarum, The Norwegian Mother and Child Cohort Study, Birth weight, Gestational age, Preterm birth, Low birthweight, Small for gestational age, Apgar score, Perinatal death, Maternal weight gain

## Abstract

**Background:**

Hyperemesis gravidarum (HG) characterized by excessive nausea and vomiting in early pregnancy, is reported to be associated with increased risks for low birthweight (LBW), preterm birth (PTB), small-for-gestational-age (SGA) and perinatal death. Conflicting results in previous studies underline the necessity to study HG’s potential effect on pregnancy outcomes using large cohorts with valid data on exposure and outcome measures, as well as potential confounders. This study aims to investigate associations between HG and adverse pregnancy outcomes using the Norwegian Mother and Child Cohort Study (MoBa).

**Methods:**

All singleton pregnancies in MoBa from 1998 to 2008 were included. Multivariable regression was used to estimate relative risks, approximated by odds ratios, for PTB, LBW, SGA and perinatal death. Linear regression was applied to assess differences in birthweight and gestational age for children born to women with and without HG. Potential confounders were adjusted for.

**Results:**

Altogether, 814 out of 71,468 women (or 1.1%) had HG. In MoBa HG was not associated with PTB, LBW or SGA. Babies born to women with HG were born on average 1 day earlier than those born to women without HG; (−0.97 day (95% confidence intervals (CI): -1.80 - -0.15). There was no difference in birthweight when maternal weight gain was adjusted for; (23.42 grams (95% CI: -56.71 - 9.86). Babies born by women with HG had lower risk for having Apgar score < 7 after 1 minute (crude odds ratio was 0.64 (95% CI: 0.43 - 0.95)). No differences between the groups for Apgar score < 7 after 5 minutes were observed. Time-point for hospitalisation slightly increased differences in gestational age according to maternal HG status.

**Conclusions:**

HG was not associated with adverse pregnancy outcomes. Pregnancies complicated with HG had a slightly shorter gestational length. There was no difference in birth weight according to maternal HG-status. HG was associated with an almost 40% reduced risk for having Apgar score < 7 after 1 minute, but not after 5 minutes. The clinical importance of these statistically significant findings is, however, rather limited.

## Background

Most women experience nausea and vomiting in early pregnancy (NVP) [1]. In contrast, hyperemesis gravidarum (HG) is a potentially life-threatening condition that occurs in between 0.8% and 3.2% [2,3]. The 10th edition of International Classification of Diseases (ICD 10) differentiates between mild (O21.0) and severe forms of HG (O21.1) [4]. The underlying mechanisms for HG remain unknown, although previous research has suggested genetic factors to be involved [5]. It is not yet clear whether maternal genes or environmental factors are the main contributing factors. Increased levels of human chorionic gonadotropin (hCG), oestrogen and leptin have been found to be associated with HG, as have increased levels of fetal DNA in maternal blood; the latter indicating damage of the feto-maternal barrier [1,6-8].

HG has been reported to be associated with low birthweight (LBW), preterm birth (PTB), small-for-gestational-age (SGA), perinatal death and prolonged stay in hospital for the new-born infant [9-12]. A recent meta-analysis on HG and pregnancy outcomes, comprising 13 case–control studies, 10 cohort studies and one cross-sectional study, reported that HG was associated with a 30% increase in risk for PTB and SGA, and a 40% increase in risk for LBW [13]. A Dutch historical cohort study among 1.2 million singleton births found HG to increase the risk for PTB by 18%, but did not find HG associated with SGA and LBW [14]. The same study, however, did report a slight difference in birthweight among children born to women with and without HG [14]. In contrast, an Canadian cohort study of 156,000 singleton pregnancies reported that women with HG gaining less than 7 kg during pregnancy had a threefold increase in risk for PTB, an almost threefold increase in risk for LBW and a fivefold increase in risk for a 5 minute Apgar score < 7 [9]. For women with HG and weight gain ≥ 7 kg, there was no increase in risk for adverse pregnancy outcomes. An American cohort study among 520,000 live births found that infants of women with HG had significantly lower birthweight and were more likely to be SGA [11].

Conflicting results in previous studies can be explained by heterogeneity of methods, definitions and confounders, in addition to the fact that most of the evidence still comes from case–control studies. Based on this, there is a need to study HG’s potential effect on pregnancy outcomes using large cohorts with valid data on both exposure and outcome measures as well as potential confounders. The aim of our study is therefore to explore associations between HG and adverse pregnancy outcomes using the Norwegian Mother and Child Cohort Study (MoBa) comprising 108,000 births.

## Methods

### Population under study

This study is a subproject in MoBa. The recruitment to MoBa occurred from 1998 to 2008. MoBa’s target population consisted of all women attending antenatal care in Norway, and the participation rate was about 40% [15]. Women were sent an invitation to participate and a written consent form simultaneously as their appointment for the routine second trimester ultrasound. Our analyses are based on three questionnaires, one received in pregnancy week 13–17 (Q1), one in week 30 (Q3) and one four to six months after birth (Q4) [16]. Q1 provided background factors, exposures and health variables. Q3 included information on health during pregnancy. In Q4 the main themes were maternal health around the birth and the time immediately after. English translations of the questionnaires can be found at http://www.fhi.no/moba. The study has been approved by the Data Inspectorate and the Regional Committee for Medical and Health Research Ethics. The birth record from the Medical Birth Registry of Norway (MBRN) is a part of the MoBa database. In Norway, notification of all live births and stillbirths from the 12th week of gestation is mandatory and information has to be sent to MBRN within one week after delivery [17]. We included singleton pregnancies only.

We excluded records with missing data on birthweight (n = 63), on gestational age (n = 349), on Apgar scores after 1 and 5 minutes (n = 194 and n = 181), on body mass index (BMI) (n = 13,556), on education (n = 4,620), and on smoking habits (n = 1,524). There was no missing data on maternal age and parity. Moreover, we also excluded children born < 22nd gestational week (n = 7) and ≥ 43rd gestational week (n = 231) and those weighing < 500 grams (n = 10) or ≥ 6000 grams (n = 7). Women with implausible values on weight and height were excluded; those reporting height < 120 cm (n = 198), missing height (n = 795) and those weighing less than 40 kg (n = 47) or more than 150 kg (n = 11) or missing weight (n = 1975) or reported weight gain during pregnancy to be > 50 kg (n = 35) or < −20 kg (n = 43). No one reported to be taller than 200 cm. The final sample consisted of 71,468 pregnancies or 78.4% of the sample.

### Variables

Given the structure of the questionnaires, the exposure variable, HG, was defined as long-lasting nausea and vomiting in pregnancy starting before the 25th gestational week and necessitating hospitalisation as reported in Q3 in MoBa. Sub-analyses have previously shown that 74% were first hospitalised during first trimester, and 25% were first admitted later in pregnancy [18]. These findings are is in line with a recent study from Sweden on HG and pregnancy outcomes based on hospital data [19].

The main outcome variables were gestational age, birthweight, LBW defined as birthweight < 2500 grams, PTB defined as gestational age < 37 gestational weeks, SGA defined as birthweight below the 10th percentile for the gestational age, perinatal death defined as death during the perinatal period (lasting from ≥ 22nd gestational week until the 7th day after birth), Apgar score after 1 minute and after 5 minutes; all obtained from MBRN.

Data on pre-pregnant BMI, parity and education were obtained from Q1. Information about maternal weight gain during pregnancy was obtained from Q1 and Q4. Data on smoking in pregnancy was obtained from Q1 and Q3, where the women registered whether they smoked on a daily basis, occasionally or did not smoke. Information on concomitant diseases such as asthma, diabetes 1, thyroid disorders, depression, anxiety, other psychological problems, anaemia, vitamin B12 deficiency and anorexia was obtained from Q1. This questionnaire included a checklist for 53 diseases or health problems, which was prefaced with ‘did you have any of these diseases or health problems before or in pregnancy?’ The diseases mentioned above were selected since previous studies have showed associations with HG [1,20,21].

Maternal age was categorised into 5 groups: younger than 20 years, 20–24 years, 25–29 years, 30–34 years and 35 years and older. By parity, women were dichotomised into nullipara and multipara. A proxy for socioeconomic status (SES) was the woman’s length of education in years categorised into 4 groups: < 12 years, 12 years, 13–16 years and ≥ 17 years. Smoking before pregnancy was categorized as no smoking and smoking including both daily and occasional smoking. BMI was grouped into 4 categories: < 18.5 kg/m^2^, 18.5-24.9 kg/m^2^, 25.0-29.9 kg/m^2^ and ≥ 30.0 kg/m^2^. Maternal weight gain during pregnancy was categorised < 0 kg, 0–6.9 kg, 7.0-14.9 kg, 15.0-19.9 kg, ≥ 20.0 kg and missing. Time-point for hospitalisation was divided into three groups: first trimester, second trimester and first-and-second trimester. Concomitant diseases, such as diabetes, Type 1 diabetes, thyroid diseases, depression, anxiety, other psychological problems, anaemia, vitamin B12 deficiency and anorexia were dichotomised into no and yes for each disease.

### Statistical analyses

Since 12,460 women contributed with more than one pregnancy, some of the data cannot be considered to be independent. Consequently, all analyses were done with generalized estimation equations (GEE) employing an independent correlation structure to obtain valid standard errors. Associations between HG and LBW, PTB, SGA and Apgar score < 7 after 1 minute and 5 minutes were studied by GEE. Crude (cOR) and adjusted (aOR) odds ratios with 95% confidence intervals (CI) were calculated. In addition, the effects of HG on birthweight and gestational age as continuous variables were studied by multiple linear regressions.

Three sets of adjustments were performed in multivariable analyses; the first included socio-demographic data as well as induction and gestational age for birthweight and Apgar score after 1 and 5 minutes. The second adjustment included concomitant medical conditions. Thirdly, we adjusted for maternal weight gain during pregnancy.

No interactions were found between HG and any other variable, suggesting no heterogeneity of the effect of HG on pregnancy outcomes. All analyses were performed using R version 2.12.1 with library geepack for GEE analyses.

## Results

Altogether, 814 out of 71,468 women (1.1%) had HG. Women with HG were younger, had less education and had to a larger extent been non-smokers before pregnancy. They had lower or higher pre-pregnancy BMI compared to those without HG. A higher proportion of women with HG gained less weight during pregnancy than those without HG (Table 1).

**Table 1:** **Prevalence of hyperemesis gravidarum (HG) with 95**% **confidence intervals (95**% **CI) according to background characteristics (N = 71,468)**

	**n (%)**	**HG (%) (95% CI)**
**Maternal age**		
< 20 years	816 (1.1)	2.8 (2.7-2.9)
20-24 years	7674 (10.7)	1.9 (1.8-2.0)
25-29 years	25267 (35.3)	1.1 (1.1-1.2)
30-34 years	27064 (37.9)	0.9 (0.9-1.0)
≥ 35 years	10647 (14.9)	1.0 (0.9-1.0)
**Parity**		
Primipara	33408 (46.8)	1.1 (1.0-1.1)
Multipara	38060 (53.2)	1.2 (1.1-1.3)
**Body mass index**		
< 18.5 (kg/m^2^)	1325 (1.9)	2.0 (1.9-2.1)
18.5-24.9 (kg/m^2^)	47390 (66.3)	1.0 (0.9-1.1)
25.0-29.9(kg/m^2^)	15727 (22.0)	1.4 (1.3-1.5)
≥ 30.0 (kg/m^2^)	7026 (9.8)	1.5 (1.4-1.6)
**Education**		
< 12 years	14168 (19.8)	1.6 (1.5-1.7)
12 years	10438 (14.6)	1.4 (1.3-1.4)
13-16 years	29569 (41.4)	1.1 (1.0-1.1)
≥ 17 years	17293 (24.2)	0.8 (0.7-0.9)
**Smoking habit**		
Non-smokers	65362 (91.5)	1.2 (1.1-1.3)
Smokers	6106 (8.5)	0.6 (0.5-0.7)
**Weight gain during pregnancy**		
< 0 kg	565 (0.8)	5.9 (5.7-6.0)
0 - 6.9 kg	3708 (5.2)	2.3 (2.2-2.5)
7.0 - 14.9 kg	26360 (36.9)	1.1 (1.1-1.2)
15.0 - 19.9 kg	15239 (21.3)	0.7 (0.7-0.8)
≥ 20 kg	8126 (11.4)	1.1 (1.0-1.1)
Missing	17470 (24.4)	1.1 (1.0-1.2)

When maternal age, parity, BMI, education, smoking habits, gestational age, induction, concomitant diseases and maternal weight gain was adjusted for, there was no difference in birth weight according HG-status (Table 2). In contrast, babies to women with HG they were on average born one day earlier compared to those born to women without HG.

**Table 2:** Differences in birthweight and gestational age for offspring of mothers with and without hyperemesis gravidarum (N = 71,468)

	**N = 71,468**	**Mean**	**Crude estimate**	**Adjusted estimate**	**Adjusted estimate**	**Adjusted estimate**
			**β [95% CI]**	β [95% CI]^*^	β [95% CI]^***^	β [95% CI]^****^
**Birthweight**						
No hyperemesis (HG)	70,654	3602 gram	1 Reference	1 Reference	1 Reference	1 Reference
HG	814	3567 gram	−34.46 (−72.84 - 3.93 )	−46.89 (−80.53 -13.26)^**^	−47.57 (−79.92- -13.21)^**^	−23.42 (−56.71- 9.86 )^**^
**Gestational age**						
No HG	70,654	279.7 days	1 Reference	1 Reference	1 Reference	1 Reference
HG	814	278.0 days	−1.68 (−2.53- -0.84 )	−1.50 (−2.34- -0.67 )	-1.38 (−2.21- -0.55)	-0.97 (−1.80- -0.15 )

^*^Adjusted for maternal age, parity, BMI, education and smoking habits.

^**^Additional adjustment for gestational age and induction.

^***^Additional adjustment for asthma, diabetes 1, thyroid disease, depression, anxiety, other psychological problems, anorexia, anaemia and vitamin B12 deficiency.

^****^Adjustment for maternal weight gain.

Slightly more than 60% of women with HG were hospitalised during first trimester only, whereas approximately 20% were admitted during second trimester. Time-point for hospitalisation did not influence birth weight or gestational age when potential confounders adjusted for (Table 3).

**Table 3:** **Differences in birth weight and gestational age with 95**% confidence intervals according to hospitalisation time-points (N = 71,425*)

	**N = 71,425**	**Mean**	**Crude estimate**	**Adjusted estimate**	**Adjusted estimate**	**Adjusted estimate**
			**β (95% CI)**	β (95% CI)**	β (95% CI)****	β (95% CI)*****
**Birth weight**						
No hyperemesis (HG)	70654	3602 gram	1	1	1	1
HG 1. Trimester	484	3591gram	−10.67 (−60.2 – 38.83)	−30.57 (−74.57 – 13.42) ^***^	−31.55 (−75.42 – 12.32) ^***^	−15.32 (−58.96 – 28.31) ^***^
HG 2. Trimester	173	3506 gram	−95.29 (−178.4 – -12.18)	−85.68 (−155.8 – -15.61) ^***^	−85.86 (−155.2 – -16.49) ^***^	−49.15 (−117.8 – 19.49) ^***^
HG 1. and 2. trimester	114	3563 gram	−39.02 (−139.9 – 61.83)	−91.20 (−176.9 – -5.48) ^***^	−92.46 (−178.2 – -6.72) ^***^	−51.06 (−138.2 – 36.11) ^***^
**Gestational age**						
No HG	70654	280 days	1	1	1	1
HG 1. Trimester	484	278 days	−1.29 (−2.31 – -0.28)	−1.12 (−2.15 – -0.10)	−1.05 (−2.07 – -0.03)	−0.79 (−1.80 – 0.23)
HG 2. trimester	173	278 days	−2.10 (−4.14 – -0.06)	−1.90 (−3.91 – 0.10)	−1.72 (−3.68 – 0.25)	−1.08 (−3.04 – 0.88)
HG 1. and 2. trimester	114	277 days	−2.56 (−4.89 – -0.24)	−2.39 (−4.70 – -0.08)	−2.22 (−4.53 – 0.09)	−1.57 (−3.89 – 0.75)

* Women with missing data on time-point for hospitalisation were excluded (n = 43).

**Adjusted for maternal age, parity, BMI, education and smoking habits.

***Additional adjustment for gestational age and induction.

****Additional adjustment for asthma, diabetes 1, thyroid disease, depression, anxiety, other psychological problems, anorexia, anaemia and vitamin B12 deficiency.

*****Adjustment for maternal weight gain.

Hyperemesis was not associated with PTB, SGA or LGA (Table 4). Children of women with HG had, however, lower risk of having Apgar score < 7 after 1 minute (cOR was 0.63 with 95% confidence interval (CI): 0.42-0.93). There was no difference in odds ratios for Apgar score < 7 after 5 minutes. Adjustment for confounders did not change these estimates. There were 167 cases of perinatal death, but none of the mothers of cases had HG. Time-point for hospitalisation due to HG did not influence the risks for adverse pregnancy outcomes (data not shown).

**Table 4:** **Risk of adverse pregnancy outcomes with 95**% **confidence intervals according to hyperemesis gravidarum (N = 71,468)**

	**n (%)**	**Prevalence (%) (95% CI)**	**Crude odds ratio (95% CI)**	Adjusted odds ratio (95% CI)^*^	Adjusted odds ratio (95% CI)^***^	Adjusted odds ratio (95% CI)^****^
**Gestational age < 37 weeks**	3157 (4.4)					
No hyperemesis (HG)		4.4 (4.3- 4.6)	1 Reference	1 Reference	1 Reference	1 Reference
HG		5.3 (5.1- 5.4)	1.21 (0.89-1.65)	1.18 (0.86-1.60)	1.15 (0.84-1.57)	1.02 (0.75-1.40)
**Birthweight < 2500 grams**	1791 (2.5)					
No HG		2.5 (2.4- 2.6)	1 Reference	1 Reference	1 Reference	1 Reference
HG		2.5 (2.3- 2.6)	0.98 (0.63-1.53)	0.80 (0.47-1.36)^**^	0.80 (0.47-1.35)^**^	0.72 (0.42-1.24)^**^
**Small for gestational age**	6793 (9.5)					
No HG		9.5 (9.3- 9.7)	1 Reference	1 Reference	1 Reference	1 Reference
HG		9.6 (9.4- 9.8)	1.01 (0.80-1.28)	1.10 (0.87-1.40)	1.11 (0.87-1.40)	1.01 (0.80-1.29)
**Apgar score after 1 minute < 7**	3553 (5.0)					
No HG		5.0 (4.8-5.2)	1 Reference	1 Reference	1 Reference	1 Reference
HG		3.2 (3.1-3.3)	0.63 (0.42-0.93)	0.62 (0.42-0.93)^**^	0.62 (0.42-0.93)^**^	0.64 (0.43-0.95)^**^
**Apgar score after 5 minutes < 7**	848 (1.2)					
No HG		1.2 (1.1-1.3)	1 Reference	1 Reference	1 Reference	1 Reference
HG		1.2 (1.1- 1.3)	1.04 (0.55-1.94)	1.07 (0.56-2.02)^**^	1.04 (0.55-1.97)^**^	1.11 (0.58-2.12)^**^
**Perinatal death**	167 (0.2)					
No HG		0.2 (0.2- 0.3)	---	---	---	---
HG		0.0 (−−)				

^*^Adjusted for maternal age, parity, BMI, education and smoking habits.

^**^Additional adjustment for gestational age and induction.

^***^Additional adjustment for asthma, diabetes 1, thyroid disease, depression, anxiety, other psychological problems, anorexia, anemia and vitamin B12 deficiency.

^****^Adjustment for maternal weight gain during pregnancy.

Women with HG gained on average 12.7 kilos during pregnancy, compared to 14.9 kilos for women without HG.

## Discussion

The main finding of our study is that HG requiring hospitalisation was not associated with increased risks for PTB, LBW or SGA. Pregnancies complicated with HG had a slightly shorter gestational length. There was no difference in birth weight according to maternal HG-status. Time-point for hospitalisation did not influence birthweight or gestational age. Moreover, HG was associated with lower risk for having Apgar score < 7 after 1 minute, whereas there was no difference in risks for Apgar < 7 score after 5 minutes. The clinical relevance of these findings is, however, limited.

### Strengths and limitations

The major strength of our study is that it has data on the main exposure and confounders from a large, nationwide pregnancy cohort. Furthermore, previous research has proven that the validity of the data in MoBa to be high [22-24]. Information on pregnancy outcomes were obtained from MBRN comprising data validity earlier described as sufficient for large scale epidemiologic research [25,26]. Altogether, the two different sets of data provide a unique opportunity to study possible effects of HG on pregnancy outcomes. To some extent MoBa suffers from selection bias as the women included are older, have more education, smoke less and are less likely to be immigrants compared to the Norwegian population at large [27]. Nilsen et al. reported in a study on MoBa and selection bias that the prevalence of conditions could be affected by selection bias, but that there was no evidence that the selection bias affected the associations studied [27]. If the validity of the observed association between HG and pregnancy outcomes should be affected by selection bias, it would require differential selection of participants related to both HG and pregnancy outcomes studied, something not considered in this study. Thus it is inconceivable that data on pregnancy outcomes from MBRN, compulsory obtained after recruitment, could be directly associated with selection to the study. Since all citizens of Norway have access to hospitals free of charge, there should be little selection to in-patient care based on economic resources of the single patient. Furthermore, as HG is associated with ethnicity, it is unfortunate that we did not have information on maternal country of birth in MoBa. However, only 5.6% of the participants reported not to have Norwegian as mother tongue. Adjustment for mother tongue did not influence our estimates.

### Comparison with other studies

Unlike the previously mentioned meta-analysis including more than 50% case–control studies, we did not find HG to be associated with an increased risk of PTB, LBW and SGA [13]. The heterogeneity described in the meta-analysis is, however, a consequence of methodological as well as clinical differences between the studies. The clinical heterogeneity was mainly related to difficulties in defining HG. The use of different diagnostic criteria may reflect that so far there is no universally accepted definition of HG, indicating that other pregnancy related conditions may have been confused with HG. Some studies had used hospital admission as a criterion, others the 8th or 9th version of the International Classification of Disease (ICD) or Fairweather’s diagnostic criteria from 1968 that included dehydration, electrolyte disturbances, ketonuria and more than 5% weight loss compared to prepregnancy weight [4,9,28]. Due to the structure of MoBa’s questionnaires, HG was in our study defined as long-lasting nausea and vomiting in pregnancy starting before the 25th gestational week which required hospitalisation. In line with previous studies, more than 70% of the women with HG in MoBa were hospitalised during the first 12 weeks [18,19]. In contrast to the recent Swedish publication, time-point for hospitalisation did not influence the estimates in our study [19]. In Norway, only women with HG and metabolic disturbances are being hospitalised, indicating that our sample includes severe HG only (or ICD 10 code O21.1). This assumption suggests that our sample was not diluted with other pregnancy related conditions, such as the more common “nausea and vomiting in pregnancy” (NVP), which up to 90% of all pregnant women suffer from [29]. However, dilution may have been a problem in the large study based on the Dutch Perinatal Registry, describing that 34% was having HG diagnoses set by midwifes in the absence of hospitalisation [14]. The prevalence of HG in the Dutch study was 0.2%, which is lower than in other European studies. The authors questioned the quality of their own dataset, suggesting an underreporting of the disease. Also; HG was associated with near to 40% increase in risk for PTB, and about 10% increase in risk for SGA [14]. When maternal characteristics, such as age, parity, ethnicity, socio-economic status and concomitant diseases were adjusted for, only PTB remained associated with HG. However, several relevant confounders, such as BMI and smoking, were not adjusted for. Another study based on Swedish births between 1973 and 1982 reported a HG prevalence of 0.3% [30]. These women were more likely to give birth before 38 gestational weeks and to deliver children with LBW. The abovementioned American cohort study among more than 500,000 live births found a HG prevalence of 0.5%, where HG was associated with SGA and LBW [11]. The two latter studies reported univariate analyses only.

In contrast, a Canadian historical birth cohort study of 156,000 pregnancies, using hospital admission before the 24th gestational week as a diagnostic criterion for HG, found a prevalence of HG of 1.0%, which is similar to our study [9]. The HG diagnosis was based on Fairweather’s diagnostic criteria, and information on most relevant confounders was available. Unlike our study, HG was found to be associated with an increased risk of PTB, LBW, SGA and Apgar score < 7 after 5 minutes, but only for women with maternal weight gain during pregnancy < 7 kg. The relative risks were 3.0, 2.8, 1.5 and 5.0, respectively. In MoBa 25% of the women in our study had missing data on maternal weight gain. Since these women had significantly higher risk of adverse pregnancy outcomes, we adjusted for maternal weight gain instead of stratifying similar to the Canadian study. Additional sub-analysis, where women with missing data on weight gain were excluded and the remaining sample was stratified according to whether the women gained less than 7 kilos or 7 kilos and more, did not change our estimates.

Maternal weight gain and body composition have, regardless of maternal HG-status, been thoroughly investigated as possible predictors for gestational age and birth weight [31,32]. A recent metanalysis of 55 studies, 37 cohort and 18 case–control including 3.5 million women, reported that low total gestational weight gain was associated with increased risks for PTB, LBW and intrauterine growth retardation (IUGR) and lower mean birth weight [31]. In this perspective, the associations between HG and adverse pregnancy outcomes reported in previous research may be explained by poor maternal weight gain rather than the mother suffering from HG [9,12]. Moreover, an American case–control study found women with HG to gain on average 4.6 kg less during pregnancy, and to deliver babies who weighed on average 291 grams less compared to those born from healthy women [10]. In MoBa women with HG gained on average 2.2 kg less than women without HG, but their babies did not have lower birth weight. However, they were born on average one day earlier. In contrast, a Norwegian institution-based case–control study reported that the 175 women hospitalised with HG gained on average 5.1 kg less than women without HG, their babies to be born 0.5 day earlier and weigh on average 138 grams less [33]. Birthweight was positively associated with maternal weight in early pregnancy, weight gain during pregnancy and parity, but not HG. This is partly in line with our study, where stepwise regression showed that differences in birth weight between babies born to women with and without HG disappeared when maternal weight gain was adjusted for. In the other study, almost 50% of the cases and more than 20% of controls had non-Norwegian names, which is different from MoBa [33]. Immigrant women in Norway are more likely to develop HG and have higher risks of adverse pregnancy outcomes such as IUGR and perinatal death [3,34,35]. It is not yet known if immigrant women with HG have more severe symptoms and gain less weight during pregnancy compared to ethnic Norwegians. Differences in results between the studies might be explained by differences in ethnic background among the women included. The results of our study may therefore not be generalisable for the total population of Norway. Since HG is not found associated with adverse pregnancy outcomes, our results might reflect good antenatal care and treatment of women hospitalised with HG participating in MoBa.

Whereas the 1 minute Apgar score reflects the immediate need for resuscitation, the 5 minute Apgar score has more a prognostic value [36]. Most studies therefore report Apgar score after 5 minutes, since this information is reckoned to be of higher clinical importance [37,38]. In our study, the children of women with HG had about 40% lower risk of Apgar score < 7 after 1 minute, but there was no difference in risk for Apgar score < 7 after 5 minutes. It is highly unlikely that this is an effect of corticosteroids administered to relieve symptoms in women with refractory HG during the first or second trimester [29,39]. Corticosteroids administered before 23rd gestational week is not known to have any effect on the fetal lung [40]. Accordingly, the 40% reduction of Apgar score in our study might reflect underlying mechanisms for HG rather than consequence of treatment. The clinical importance of this statistical significant finding, however, is considered limited.

Although HG in our study did not have any negative short-term consequences for the offspring, the possibility for long-term consequences have barely been studied. Previous research has shown that metabolic changes in women with HG might resemble those resulting from starvation [41]. Given the fact that fetal undernutrion during first trimester is associated with cardiovascular disease, diabetes and schizophrenia in later life, HG may also influence disease patterns [41,42].

## Conclusions

In MoBa HG requiring hospitalisation was not associated with adverse pregnancy outcomes. Pregnancies complicated with HG had slightly shorter gestational length. However, there was no difference in birth weight according to maternal HG-status. Additionally, HG was associated with a decreased risk for delivering a child with Apgar score < 7 after 1 minute, but no difference was observed after 5 minutes. The clinical relevance of these statistically significant findings is rather limited, other than comforting the mothers-to-be by informing them that HG does not seem to increase the risk for PTB or having a child with LBW and SGA or low Apgar scores.

## Abbreviations

95% CI: 95% confidence interval; HG: Hyperemesis gravidarum; LBW: Low birth weight; MBRN: Medical Birth Registry of Norway; MoBa: The Norwegian Mother and Child Cohort Study; OR: Odds ratio; PTB: Preterm birth; SGA: Small for gestational age.

## Competing interest

The authors declare that they have no competing interest.

## Authors’ contributions

ÅV, AMG, NCS and PM designed the study. ÅV, NCS and AMG analysed the data, whereas ÅV, AMG, NCS and PM all contributed to the interpretation of the data. ÅV, AMG and NCS drafted the article, and PM revised critically for important intellectual content. All authors contributed to the writing process. All authors read and approved the final manuscript.

## Pre-publication history

The pre-publication history for this paper can be accessed here:

http://www.biomedcentral.com/1471-2393/13/169/prepub
